# Role of Bacterial Surface Structures on the Interaction of *Klebsiella pneumoniae* with Phagocytes

**DOI:** 10.1371/journal.pone.0056847

**Published:** 2013-02-15

**Authors:** Catalina March, Victoria Cano, David Moranta, Enrique Llobet, Camino Pérez-Gutiérrez, Juan M. Tomás, Teresa Suárez, Junkal Garmendia, José A. Bengoechea

**Affiliations:** 1 Laboratory Microbial Pathogenesis, Fundació d'Investigació Sanitària de les Illes Balears (FISIB), Bunyola, Spain; 2 Program Host-Pathogen Interactions, Centro de Investigación Biomédica en Red Enfermedades Respiratorias (CibeRes), Bunyola, Spain; 3 Departamento de Microbiología, Facultad de Biología, Universidad de Barcelona, Barcelona, Spain; 4 Departamento de Medicina Celular y Molecular, Centro de Investigaciones Biológicas (CSIC), Madrid, Spain; 5 Instituto de Agrobiotecnología, CSIC-Universidad Pública de Navarra-Gobierno de Navarra, Mutilva, Spain; 6 Consejo Superior de Investigaciones Científicas (CSIC), Madrid, Spain; Université d'Auvergne Clermont 1, France

## Abstract

Phagocytosis is a key process of the immune system. The human pathogen *Klebsiella pneumoniae* is a well known example of a pathogen highly resistant to phagocytosis. A wealth of evidence demonstrates that the capsule polysaccharide (CPS) plays a crucial role in resistance to phagocytosis. The amoeba *Dictyostelium discoideum* shares with mammalian macrophages the ability to phagocytose and kill bacteria. The fact that *K. pneumoniae* is ubiquitous in nature and, therefore, should avoid predation by amoebae, poses the question whether *K. pneumoniae* employs similar means to counteract amoebae and mammalian phagocytes. Here we developed an assay to evaluate *K. pneumoniae*-*D. discoideum* interaction. The richness of the growth medium affected the threshold at which the *cps* mutant was permissive for *Dictyostelium* and only at lower nutrient concentrations the *cps* mutant was susceptible to predation by amoebae. Given the critical role of bacterial surface elements on host-pathogen interactions, we explored the possible contribution of the lipopolysaccharide (LPS) and outer membrane proteins (OMPs) to combat phagoyctosis by *D. discoideum*. We uncover that, in addition to the CPS, the LPS O-polysaccharide and the first core sugar participate in *Klebsiella* resistance to predation by *D. discoideum*. *K. pneumoniae* LPS lipid A decorations are also necessary to avoid predation by amoebae although PagP-dependent palmitoylation plays a more important role than the lipid A modification with aminoarabinose. Mutants lacking OMPs OmpA or OmpK36 were also permissive for *D. discoideium* growth. Except the LPS O-polysaccharide mutants, all mutants were more susceptible to phagocytosis by mouse alveolar macrophages. Finally, we found a correlation between virulence, using the pneumonia mouse model, and resistance to phagocytosis. Altogether, this work reveals novel *K. pneumoniae* determinants involved in resistance to phagocytosis and supports the notion that *Dictyostelium* amoebae might be useful as host model to measure *K. pneumoniae* virulence and not only phagocytosis.

## Introduction

Phagocytosis is the process by which particles are recognized, bound to the surface of cells and internalized into a plasma membrane-derived intracellular vacuole, or phagosome. In mammals, phagocytosis is a special feature of the so-called professional phagocytes, i.e. polymorphonuclear leukocytes (also known as neutrophils), dendritic cells, monocytes and macrophages. When a microorganism enters the sterile sections of the body, professional phagocytes are chemotactically attracted, bind the microorganism, ingest and kill it. In the case of macrophages and dendritic cells, the invader's antigenic molecules are presented to other immune cells hence initiating adaptive immune responses.

The social amoeba *Dictyostelium discoideum* lives in soil, where it feeds on both Gram-negative and positive bacteria [1]. Typically, upon starvation, *Dictyostelium* cells initiate a multicellular development stage leading to the formation of a fruit body. However, there are *D. discoideum* axenic strains that can feed not only by phagocytosis but also by macropynocytosis of liquid nutrients [2]. Of special interest is the fact that *Dictyostelium* cytoskeleton architecture is similar to that found in mammalian cells. Furthermore, the process of particle uptake in *Dictyostelium* is similar to macrophage phagocytosis [1]. The fact that the strategies evolved to counteract mammalian professional phagocytes are considered essential to establish an infection has led to the notion that *Dictyostelium* amoebae could be used as host model to measure virulence [3], [4]. The similarities between *Dictyostelium* and mammalian cells also extent to membrane trafficking, endocytic transport and sorting events [1]. There are genetic tools available to manipulate *D. discoideum* cells thereby facilitating the study of cellular mechanisms at the molecular level. Moreover, the genome of *D. discoideum* strain AX4 has been sequenced [5].

*Klebsiella pneumoniae* is a Gram negative pathogen common cause of nosocomial infections that include urinary tract, respiratory, and wound infections [6]. *K. pneumoniae* isolates are frequently resistant to multiple antibiotics [7], which leads to a therapeutic dilemma. In contrast to many bacterial pathogens, *K. pneumoniae* is ubiquitous in nature. The non-clinical habitats include the mucosal surfaces of animals and environmental sources such as vegetation, soil and surface waters [8]. Notably, it has been shown that environmental *Klebsiella* isolates are nearly identical to clinical ones with respect to the expression of virulence factors and ability to infect animal models [9]. The factors mediating *Klebsiella* survival in the environment are poorly characterized but predominance in the environment is likely to correlate with the ability of *Klebsiella* to avoid predation by protozoa, including amoebae.

Macrophages and neutrophils play a critical role in the clearance of bacteria from the lung and other organs by their capacity for phagocytosis and killing. In this regard, it has been shown that depletion of either neutrophils or alveolar macrophages results in reduced killing of *K. pneumoniae in vivo* [10], [11]. Conversely, this suggests that *Klebsiella* countermeasures against phagocytosis should be important virulence factors. Supporting this notion, *K. pneumoniae* capsule (CPS) reduces phagocytosis by neutrophils and macrophages [12]–[14] and CPS mutant strains are avirulent being not able to cause pneumonia and urinary tract infections [13], [15], [16]. Notably, CPS is also important to prevent phagocytosis by *D. discoideum* [17]–[19]. Therefore, a tantalizing hypothesis could be that *K. pneumoniae* may employ the same determinants for resistance to phagocytosis by neutrohils, macrophages and amoebae. Moreover, given the critical role of bacterial surface elements on host-pathogen interactions, we speculated that the lipopolysaccharide (LPS) and outer membrane proteins (OMPs), major components of the outer membrane (OM) of Gram negative bacteria, could be also involved in the resistance to phagocytosis by *K. pneumoniae*.

LPS consists of a hydrophobic membrane anchor, lipid A, substituted with an oligosaccharide core region that can be extended in some bacteria, including *Klebsiella*, by a repeating oligosaccharide, the O-polysaccharide (OPS). The LPS contains a molecular pattern recognized by the innate immune system thereby arousing several host defence responses. The lipid A could be decorated with aminoarabinose, palmitate or phosphoethanolamine [20]. Several studies have demonstrated that these modifications are involved in the resistance to antimicrobial peptides, key weapons of the innate immune system against infections [21]–[25]. In a recent study we have shown that *K. pneumoniae* lipid A is decorated with palmitate and aminoarabinose which contribute to *K. pneumoniae* resistance to antimicrobial peptides [26]. OMPs are important for membrane integrity and transport of molecules across (for a review see [27]). OmpA is one of the best characterized OM protein and data support the notion that plays an important role in the interaction of bacteria with the innate immune system (for a review see [28]). OmpA and OmpK36 are the most abundant OMPs on *K. pneumoniae* OM [29]. Mounting evidence indicates that *K. pneumoniae* OmpA is important for immune evasion *in vitro* and *in vivo* [30], [31].

In this study, we report that *K. pneumoniae* employs the same determinants to counteract phagocytosis by *D. discoideum* and alveolar macrophages, the resident defenders of the lung against infections. We uncover that the LPS OPS, the first LPS core sugar, the lipid A decorations with palmitate and aminoarabinose, and the OMPs OmpA and OmpK36 contribute to the resistance to phagoyctosis by *D. discoideum* and alveolar macrophages. Finally, we report a correlation between virulence, using the pneumonia mouse model, and resistance to phagocytosis.

## Materials and Methods

### Ethics statement

Mice were treated in accordance with the European Convention for the Protection of Vertebrate Animals used for Experimental and other Scientific Purposes (Directive 86/609/EEC) and in agreement with the Bioethical Committee of the University of the Balearic Islands. This study was approved by the Bioethical Committee of the University of the Balearic Islands with the authorisation number 1748.

### Bacterial strains and growth conditions

Bacterial strains and plasmids used in this study are listed in Table 1. Strains were grown in lysogeny broth (LB) at 37°C on an orbital shaker (180 rpm). When appropriate, antibiotics were added to the growth medium at the following concentrations: rifampicin (Rif) 25 µg/ml, ampicillin (Amp), 100 µg/ml for *K. pneumoniae* and 50 µg/ml for *E. coli*; kanamycin (Km) 100 µg/ml; chloramphenicol (Cm) 12.5 µg/ml.

**Table 1:** Strains and plasmids used in this study.

Bacterial strains and plasmids	Genotype or comments	Source or references
**Strains**		
*Escherichia coli*		
C600	*Thi, thr, leuB, tonA, lacY, supE*	[61]
CC118-λpir	Δ(*ara-leu*)7697 *araD139* Δ*lacX74 galE galK* Δ*phoA20 thi-1 rpsE rrpoB argE*(Am) *recA1*	
*Klebsiella pneumoniae*		
Kp52145	clinical isolate (serotype O1:K2), Rif^R^	[13], [62]
52145-Δ*wca_K2_*	Kp52145, Δ*wcaK2*; the *wca_K2_* gene inactivated, no CPS expression; Rif^R^	[63]
52145-Δ*pmrF*	Kp52145, Δ*pmrF*; the *pmrF* gene inactivated; nonpolar mutant; Rif^R^	[26]
52145-Δ*pagP*GB	Kp52145, Δ*pagP*::Km-GenBlock; the *pagP* gene inactivated; nonpolar mutant; Rif^R^, Km^R^	[26]
52145-Δ*wca_K2_*-Δ*pmrF*	52145-Δ*wca_K2_*, Δ*pmrF*; the *pmrF* gene inactivated in CPS mutant background; Rif^R^, Km^R^	[26]
52145-Δ*wca_K2_*-Δ *pagP*GB	52145-Δ*wca_K2_*, Δ*pagP*::Km-GenBlock; the *pagP* gene inactivated in CPS mutant background; Rif^R^, Km^R^	[26]
52145-Δ*pagP*GB-Δ*pmrF*	52145-Δ*pagP*GB, Δ*pmrF*; the *pmrF* gene inactivated in *pagP* mutant background; Rif^R^, Km^R^	This work
52145-Δ*wca_K2_*-Δ*pagP*GB-Δ*pmrF*	52145-Δ*wca_K2_*-Δ*pagP*GB, Δ*pmrF*; the *pmrF* gene inactivated in *cps*-*pagP* mutant background; Rif^R^, Km^R^	This work
52OmpA2	Kp52145, *ompA* gene inactivated by insertion of pKNOCKIntKpnOmpA; Rif^R^, Cm^R^	[30]
52145-Δ*wca*_K2_-*ompA*	52145-Δ*wca_K2_*; *ompA* gene inactivated by insertion of pKNOCKIntKpnOmpA; Rif^R^, Cm^R^	[30]
52OmpA2Com	Kp52145 *ompA* mutant harbouring mini-Tn7TKmKpnOmpA; OmpA levels restored; Rif^R^, Cm^R^, Km^R^	[30]
52145- Δ*wca*_K2_-*ompA*Com	52145-Δ*wca_K2_ ompA* mutant harbouring mini-Tn7TKmKpnOmpA; OmpA levels restored; Rif^R^, Cm^R^, Km^R^	[30]
52OmpK36	Kp52145, *ompK36* gene inactivated by insertion of pKNOCKIntKpnOmpK36; Rif^R^, Cm^R^	[30]
52145-Δ*wca*_K2_-*ompK36*	52145-Δ*wca_K2_*; *ompA* gene inactivated by insertion of pKNOCKIntKpnOmpA; Rif^R^, Cm^R^	This work
52OmpK36Com	Kp52145 *ompK36* mutant harbouring mini-Tn7TKmKpnOmpK36; OmpK36 levels restored; Rif^R^, Cm^R^, Km^R^	This work
52145-Δ*wca*_K2_-*ompK36*Com	52145-Δ*wca_K2_ ompK36* mutant harbouring mini-Tn7TKmKpnOmpK36; OmpK36 levels restored; Rif^R^, Cm^R^, Km^R^	This work
52O21	Kp52145, *wbbM* gene inactivated; Rif^R^, Km^R^	[13]
52145-Δ*waaL*	Kp52145; Δ*waaL*; the *waaL* gene inactivated; nonpolar mutant; Rif^R^	[34]
52145-Δ*wca_K2_*-Δ*waaL*	52145-Δ*wca_K2_*, Δ*waaL*; the *waaL* gene inactivated; nonpolar mutant; Rif^R^	This work
52145-Δ*wabM*	Kp52145; Δ*wabM*; the *wabM* gene inactivated; nonpolar mutant; Rif^R^	[35]
52145-Δ*wabH*	Kp52145; Δ*wabH*; the *wabH* gene inactivated; nonpolar mutant; Rif^R^	[35]
52145-Δ*wabK*	Kp52145; Δ*wabK*; the *wabK* gene inactivated; nonpolar mutant; Rif^R^	[35]
52145-Δ*wabG*	Kp52145; Δ*wabG*; the *wabG* gene inactivated; nonpolar mutant; Rif^R^	[34]
52145-Δ*waaQ*	Kp52145; Δ*waaQ*; the *waaQ* gene inactivated; nonpolar mutant; Rif^R^	[52]
52145-Δ*waaL-*Δ*waaQ*	52145-Δ*waaL*, Δ*waaQ*; the *waaQ* gene inactivated; nonpolar mutant; Rif^R^	[52]
52145-Δ*wca_K2_*-Δ*wabM*	52145-Δ*wca_K2_*, Δ*wabM*; the *wabM* gene inactivated; nonpolar mutant; Rif^R^	This work
52145-Δ*wca_K2_*-Δ*wabH*	52145-Δ*wca_K2_*, Δ*wabH*; the *wabH* gene inactivated; nonpolar mutant; Rif^R^	This work
52145-Δ*wca_K2_*-Δ*wabK*	52145-Δ*wca_K2_*, Δ*wabK*; the *wabK* gene inactivated; nonpolar mutant; Rif^R^	This work
**Plasmids**		
pGEM-T Easy	Cloning plasmid, Amp^R^	Promega
pKO3	Suicide vector, Psc101 replication origin, *sacB* gene, Cm^R^	[32]
pKOV	pKO3 with the addition of a 3 kb stuffer sequence in the multiple cloning site; Cm^R^	Addgene plasmid 25769
pGEMTΔ*pmrF*	pGEM-T Easy containing Δ*pmrF*; Amp^R^	[26]
pKOVΔ*pmrF*	pKOV containing Δ*pmrF*; Cm^R^	This study
pKO3Δ*waaL*	pKO3 containing engineered *waaL* deletion; Cm^R^	[34]
pKO3Δ*wabM*	pKO3 containing engineered *wabM* deletion; Cm^R^	[35]
pKO3Δ*wabH*	pKO3 containing engineered *wabH* deletion; Cm^R^	[35]
pKO3Δ*wabK*	pKO3 containing engineered *wabK* deletion; Cm^R^	[35]
pKNOCKIntKpnOmpK36	pKNOCK-Cm containing an internal fragment from *ompK36*; Cm^R^.	[30]
pUC18R6KT-mini-Tn7TKm	pUC18R6KT-mini-Tn7T containing a Km cassette; Amp^R^, Km^R^	[30]
pUC18R6KT-mini-Tn7TKmKpnOmpK36	pUC18R6KT-mini-Tn7TKm; 1.7 kb encompassing *ompK36* and its promoter; Amp^R^, Km^R^	This work
pFPV25.1	*rpsM::gfpmut3*; Amp^R^	[39]
pFPV25.1Cm	pFPV25.1; *cat* cassette cloned into EcoRV site; Amp^R^, Cm^R^	This work

### Construction of *K. pneumoniae* mutants

To construct a *pmrF* mutant, pGEMT*pmrF* was amplified by inverse PCR to delete internal coding regions of *pmrF* using primers KpnpmrFinvF and KpnpmrFinvR (Table 2). The PCR product was digested with DpnI, gel purified and ligated to obtain pGEMTΔ*pmrF*. Δ*pmrF* allele was PCR- amplified using *Vent* polymerase and primers KpnpmrFF and KpnpmrFR (Table 2), and cloned into SmaI-digested pKOV [32] to obtain pKOVΔ*pmrF*. This vector was electroporated into 52145-Δ*pagP*GB and 52145-Δ*wca*_K2_-Δ*pagP*GB and clones were selected after growth on LB agar plates supplemented with Cm at 30°C. Bacteria from 10 individual colonies were pooled in 500 Δl PBS, serially diluted in PBS, and spread on LB agar plates with Cm which were incubated at 42°C in order to select merodiploids in which the suicide vector was integrated into the chromosome by homologous recombination. 5–10 merodiploids were serially diluted in PBS and dilutions spread in LB agar plates containing 10% sucrose and without NaCl which were incubated at 30°C. The recombinants that survived 10% sucrose were checked for their antibiotic resistance. The replacement of the wild-type alleles by the mutant ones was confirmed by PCR (data not shown). Recombinants selected were named 52145-Δ*pagP*GB-Δ*pmrF* and 52145-Δ*wca*_K2_-Δ*pagP*GB-Δ*pmrF*.

**Table 2:** Primers used in this study.

Sequences of primers used in this study		
Purpose/arget gene	Name	Sequence (5′ to 3′)
**Mutagenesis**		
	KpnpmrFF	CGGATCCACCTGCGCGAGCTGGCGGAC
* pmrF*	KpnpmrFR	CGGATCCCGGCGTCATCCGCGCCAATC
	KpnpmrFinvF	TCTCCTCCGGCGGGTTTTGC
	KpnpmrFinvR	CAAATACAGCTTTATGCGCCTG
**Complementation**		
*ompK36*	ComKpnOmpK36F	GGAGTGGTAGCTGAATCGCAGC
	ComKpnOmpK36R	AGGGAATCATTAGCCGTAGCAC
**RT-qPCR**		
*pmrI*	KpnyfbGF1	CGCTGGATCTACTCGGTCTC
	KpnyfbGR1	TCTTTGTTCTCGATGATGCG
*rpoD*	KpnrpoDLEFT	CCGGAAGACAAAATCCGTAA
	KpnrpoDRIGHT	CGGGTAACGTCGAACTGTTT
**Tn7 insertion**		
*glmS*	KpnglmSup	GCGACAACTGTTGCGACGGTG
	KpnglmSdown	TGGCTTATCACGTCGCGCTG
Tn7	Ptn7L	ATTAGCTTACGACGCTACACCC
	Ptn7R	CACAGCATAACTGGACTGATTTC

To confirm that *pmrF* mutation does not have polar effects, the expression of the downstream gene, *pmrI*, was analyzed by real time quantitative PCR (RT-qPCR). Bacteria were grown in 5 ml of LB on an orbital incubator shaker (180 r.p.m.) until an OD_600_ of 0.3. 0.5 ml of ice-cold solution EtOH/phenol [19∶1 v/v (pH 4.3)] were added to the culture and the mixture was incubated on ice for 30 min to prevent RNA degradation. Total RNA was extracted using a commercial NucleoSpin RNA II kit as recommended by the manufacturer (Macherey-Nagel). cDNA was obtained by retrotranscription of 2 µg of total RNA using a commercial M-MLV Reverse Transcriptase (Sigma), and random primers mixture (SABiosciences, Quiagen). 200 ng cDNA were used as a template in a 25 µl reaction mixture containing 1x SYBR green RT^2^ qPCR Master Mix (Superarray Bioscience Corporation) and primer mix (KpnyfbGF1 and KpnyfbGR1). *rpoD* was amplified as control using primers KpnrpoDLEFT and KpnrpoDRIGHT (Table 2). RT-qPCR analyses were performed as previously described [33]. The expression of *pmrI* was not significantly different between strains (data not shown).

To obtain *K. pneumoniae* mutant strains with defects in LPS core, chromosomal in-frame nonpolar *waa* deletions were generated [34], [35]. pKO3Δ*wabM*, pKO3Δ*wabH*, pKO3Δ*wabK* suicide vectors were used to introduce each mutation into the *cps* mutant, strain 52145-Δ*wca*_K2_, by double homologous recombination, as previously described [34], [35]. Likewise, a double mutant lacking *cps* and OPS was constructed by mobilizing the suicide vector pKO3Δ*waaL* into 52145-Δ*wca*_K2_.

An *ompK36* mutant in the genetic background of the *cps* mutant, strain 52145-Δ*wca*_K2_, was obtained by insertion-duplication mutagenesis using the suicide vector pKNOCKIntKpnOmpK36. Correct insertion was verified by Southern blot (data not shown). OMPs were purified and analyzed by SDS-PAGE using 12% polyacrylamide gels as previously described [30], [36]. Proteins were visualized by Coomassie brilliant blue staining. 52145-Δ*wca*_K2_-Δ*ompK36* did not express OmpK36 whereas the expression of other OMPs was not affected (Figure S1).

### Complementation *ompK36* mutants

A 1.7 kb fragment encompassing *ompK36* and its promoter was PCR-amplified (using primers ComKpnOmpK36F and ComKpnOmpK36R [Table 2], *Vent* polymerase [New England Biolabs]) and cloned into SmaI-digested pUC18R6KT-mini-Tn7TKm to give pUC18R6KT-mini-Tn7TKmKpnOmpK36. Tn7 delivery to 52OmpK36 and 52145-Δ*wca*_K2_-Δ*ompK36* was performed as described [37] and insertion was verified by colony-PCR with primer pairs: KpnglmSup/Ptn7L; and KpnglmSdown/Ptn7R [37]. Tn*7* transposon integrates at the site-specific *att*Tn*7*, located downstream of the *glmS* gene, thereby introducing *ompK36* gene under the control of its own promoter into the chromosome. The complemented strains, 52OmpK36Com and 52145-Δ*wca*_K2_-Δ*ompK36*Com, expressed amounts of OmpK36 similar to those of the wild-type strain (Figure S1).

### Construction of pFPV25.1Cm plasmid

A *cat* cassette, obtained by SmaI digestion of p34S-Cm [38], was cloned into EcoRV-digested pFPV25.1 [39], [40] to obtain pFPV25.1Cm. This plasmid expresses *gfpmut3* under the control of *Salmonella rpsM* promoter region. This fusion has been reported to be expressed at similar levels in various environments, including growth media and mammalian cells [39], [40]. pFPV25.1Cm was introduced into *E. coli* CC118-λ*pir* from which it was mobilized into *Klebsiella* strains by triparental conjugation using the helper strain *E. coli* HB101/pRK2013.

### LPS analysis

Small scale LPS extraction using hot phenol was performed following the procedure described by Marolda *et al*. [41], with the exception that ethyl ether was replaced by ethanol for the washing of the LPS pellet. The LPS was run on a 12% SDS-PAGE and visualized using Pro-Q Emerald 300 Lipopolysaccharide Gel Stain Kit (Invitrogen).

### Eukaryotic cells culture

*D. discoideum* AX2 cells were grown at 21°C in HL5 medium (pH 6.5) supplemented with 1.12 mg/ml glucose, 20 µg/ml streptomycin and 10 µg/ml tetracycline, and subcultured twice a week to maintain a density <10^6^ cells/ml [42], [43].

Murine alveolar macrophages MH-S (ATTC, CRL-2019) were grown on RPMI 1640 tissue culture medium supplemented with 10% heat-inactivated fetal calf serum (FCS) and Hepes 10 mM at 37°C in an humidified 5% CO_2_ atmosphere.

### Growth of *Dictyostelium* on bacteria

Procedures to test growth of *Dictyostelium* on bacteria have been described previously [42]. Briefly, bacteria were grown overnight in 5-ml LB, harvested (2500× *g*, 20 min, 24°C), washed once with PBS and a suspension containing approximately 1×10^9^ cfu/ml was prepared in 10 mM PBS (pH 6.5). 300 µl from this suspension was spread onto standard medium (SM)-agar plates (10 g/l glucose, 10 g/l peptone, 1 g/l yeast extract, 1 g/l MgSO_4_:7H_2_O, 1.9 g/l KH_2_PO_4_, 0.6 g/l K_2_HPO_4_, 20 g/l agar; pH 6.3) or dilution series of HL5-agar plates. The plates were dried in a laminar hood for 30 min. Variable numbers of *Dictyostelium* amoebae (10 000, 1000, 100, 10) were deposited on the bacterial lawn, and allowed to grow at 21°C for 4–5 days, i.e. until *Dictyostelium* growth became visible.

### Phagocytosis and killing of bacteria by *Dictyostelium*

Experiments were performed as previously described [44]. Briefly, bacteria were grown in 5-ml LB, harvested in the exponential phase (2500x *g*, 20 min, 24°C), washed once with PBS and a suspension containing approximately 1×10^9^ cfu/ml was prepared in 10 mM PBS (pH 6.5). To test the ability of *Dictyostelium* to ingest and kill live bacteria, 10^4^ cfu from the indicated suspension were mixed with 10^6^ *Dictyostelium* in 500 µl of KK2 buffer (16.5 mM KH_2_PO_4_, 3.9 mM K_2_HPO_4_; pH 6.3) and incubated at 21°C with shaking. After 90 or 180 min of incubation, a 10 µl aliquot of the suspension was collected and diluted in 40 µl of ice-cold sucrose (400 g/l). 200 µl of 0.5% saponin in KK2 were added, before plating on a LB agar plate and incubating at 37°C. Control experiments showed that this procedure does not affect bacterial viability (this work and [44]). When indicated, the number of viable bacteria associated with *Dictyostelium* cells (intracellular fraction) was determined by washing the cells twice with ice-cold HL5 medium before diluting in sucrose [44]. Results are expressed as percentage of the colony count of bacteria not exposed to *D. discoideum*. All experiments were done with triplicate samples on at least four independent occasions.

Immunofluorescence analysis was performed as described previously [45] by infecting AX2/RPF, a *D. discoideum* strain constitutively expressing the red fluorescent protein [46]. 2.5×10^7^ cells were seeded on 12 mm circular coverslips in 24-well tissue culture plates and, after 2 h, infected with GFP-expressing *K. pneumoniae* at a ratio of 100 bacteria per 1 cell in a final volume of 500 µl of HL5. To synchronize infection, plates were centrifuged at 200× *g* during 5 min. Plates were incubated at 21°C for 30 min. Cells were washed two times with KK2 buffer and fixed with 3.7% paraformaldehyde in PBS pH 7.4 for 20 min at room temperature. Coverslips were washed two times in KK2 buffer before mounting onto glass slides using Aqua poly/Mount (Polysciences). Confocal microscopy was carried out with a Leica TCS SP5 confocal microscope. Experiments were carried out by duplicate in three independent occasions. The number of infected cells and the number of intracellular bacteria per cell was quantified within 300 cells.

### Killing of *Dictyostelium* by bacteria

Bacteria were grown in 5-ml LB, harvested in the exponential phase (2500× *g*, 20 min, 24°C), washed once with PBS and a suspension containing approximately 1×10^9^ cfu/ml was prepared in 10 mM PBS (pH 6.5). To test the ability of *Klebsiella* to kill *Dictyostelium*, 10^4^ cfu were mixed with 10^6^ *Dictyostelium* cells in 500 µl of KK2 buffer and incubated at 21°C with shaking (180 rpm). After 3 h, serial dilutions of the mixture were plated on a *K. aerogenes* lawn on SM agar plates, which were incubated at 21°C for 4–5 days, i.e. until individual colonies of *Dictyostelium* became visible. All experiments were done with triplicate samples on three independent occasions.

### Phagocytosis of bacteria by alveolar macrophages

MH-S cells were seeded in 24-well tissue culture plates at a density of 7×10^5^ cells per well 15 h before the experiment. Bacteria were grown in 5-ml LB, harvested in the exponential phase (2500× *g*, 20 min, 24°C), washed once with PBS and a suspension containing approximately 1×10^9^ cfu/ml was prepared in 10 mM PBS (pH 6.5). Cells were infected with 35 µl of this suspension to get a multiplicity of infection of 50∶1 in a final volume of 500 µl RPMI 1640 tissue culture medium supplemented with 10% heat-inactivated FCS and 10 mM Hepes. To synchronize infection, plates were centrifuged at 200× *g* during 5 min. Plates were incubated at 37°C in an humidified 5% CO_2_ atmosphere. After 30 min of contact, cells were washed twice with PBS and incubated for additional 90 min with 500 µl RPMI 1640 containing 10% FCS, 10 mM Hepes, gentamicin (300 µg/ml) and polymyxin B (15 µg/ml) to eliminate extracellular bacteria. This treatment did not induce any cytotoxic effect which was verified measuring the release of lactate dehydrogenase (LDH) and by immunofluorescence microscopy (data not shown). Cells were then washed three times with PBS and lysed with 300 µl of 0.5% saponin in PBS for 10 min at room temperature. Serial dilutions were plated on LB to quantify the number of intracellular bacteria. Phagocytosis data are represented as cfu per well. All experiments were done with triplicate samples on at least three independent occasions.

Immunofluorescence was performed as previously described [47]. Cells were seeded on 12 mm circular coverslips in 24-well tissue culture plates. Infections were carried out as described before with *K. pneumoniae* strains harbouring pFPV25.1Cm. After 90 min, cells were washed three times with PBS, and fixed with 3.7% paraformaldehyde in PBS pH 7.4. The actin cytoskeleton was stained with Rhodamine-Phalloidin (Invitrogen) diluted 1∶100, DNA was stained with Hoescht 33342 (Invitrogen) diluted 1∶2500. Staining was carried out in 10% horse serum, 0.1% saponin in PBS. Coverslips were washed twice in PBS containing 0.1% saponin, once in PBS, and incubated for 30 minutes with primary antibodies. Coverslips were then washed twice in 0.1% saponin in PBS and once in PBS and incubated for 30 minutes with secondary antibodies. Finally, coverslips were washed twice in 0.1% saponin in PBS, once in PBS and once in H_2_O, mounted on Aqua Poly/Mount (Polysciences) and analysed with a Leica TCS SP5 confocal microscope. The number of infected cells and the number of intracellular bacteria per cell was quantified within 300 cells. Experiments were carried out by triplicate in three independent occasions.

### Intranasal infection model

Five- to 7-week-old female C57BL/6JOlaHsd mice (Harlan) were anesthetized by intraperitoneal injection with a mixture containing ketamine (50 mg/kg) and xylazine (5 mg/kg). Overnight bacterial cultures were centrifuged (2500× *g*, 20 min, 24°C), resuspended in PBS and adjusted to 5×10^4^ cfu/ml. 20 µl of the bacterial suspension were inoculated intranasally in four 5 µl aliquots. To facilitate consistent inoculations, mice were held vertically during inoculation and placed on a 45° incline while recovering from anaesthesia. At indicated times after infection, mice were euthanized by cervical dislocation and lungs were rapidly dissected for bacterial loads determination. Dissected lungs were homogenized in 500 µl of PBS using an Ultra-Turrax TIO basic (IKA) on ice. Serially diluted bacteria from the homogenates were recovered in LB agar plates containing Rif for wild-type strain or Cm for *ompK36* mutant. Results are reported as log cfu per gram of tissue.

### Statistical analysis

Statistical analyses were performed using the two-tailed *t* test or, when the requirements were not met, by the Mann-Whitney U test. *P*<0.05 was considered statistically significant. The analyses were performed using Prism4 for PC (GraphPad Software).

## Results

### Role of *K. pneumoniae* CPS on phagocytosis resistance

**Figure 1:**
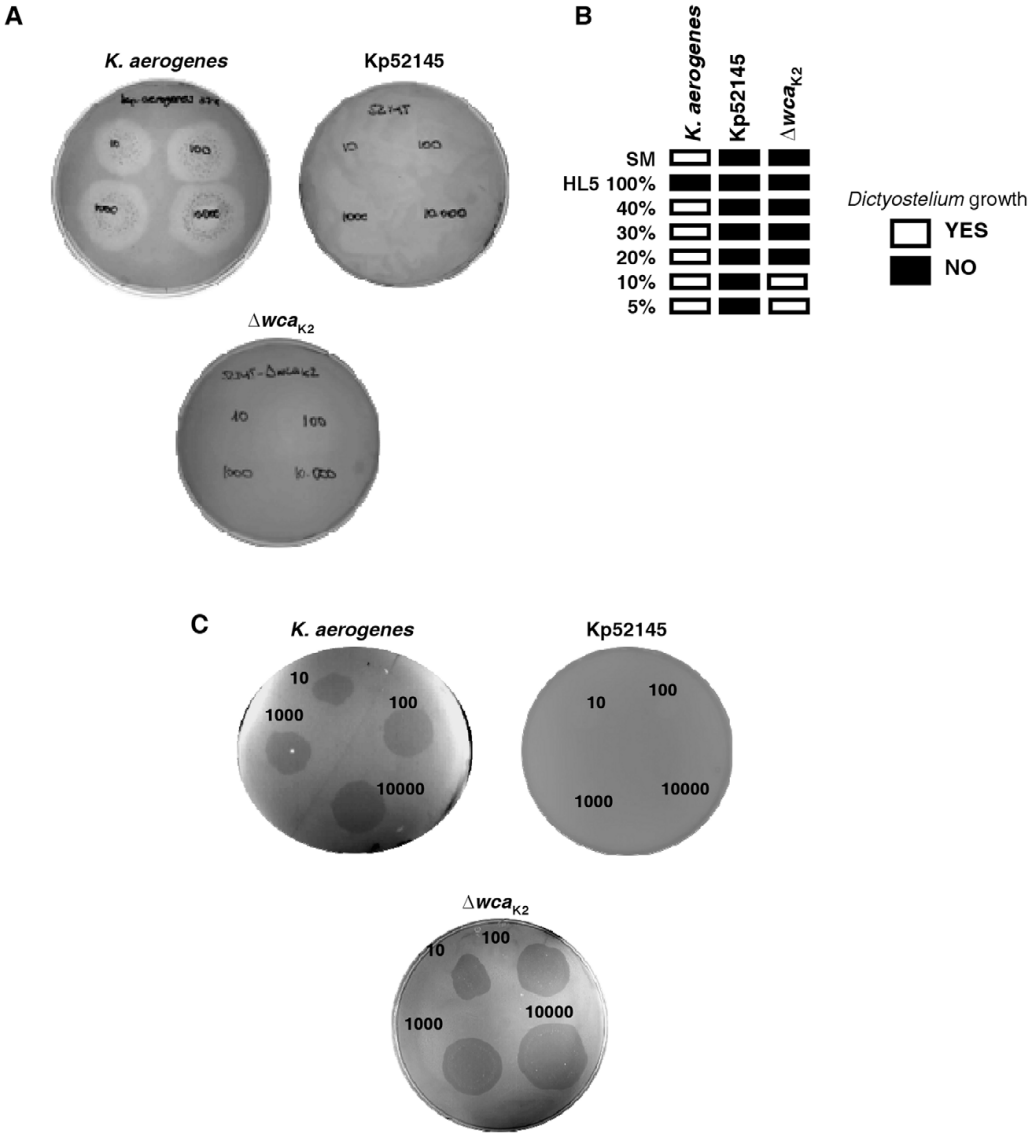
Virulence of *K. pneumoniae* against *D. discoideum* can be modulated. (A) The ability of *Dictyostelium* to grow on a bacterial lawn was assessed by depositing amoebae (from 10 to 10,000) on a lawn of bacteria grown on SM agar medium. A phagocytosis plaque was observed 5 days later when bacteria were permissive. Bacteria tested were: *K. pneumoniae* (Kp52145), *cps* mutant (52145-*Δwca*_K2_; *Δwca*_K2_), or control strain (*K. aerogenes*). (B) The ability of wild-type *K. pneumoniae* (Kp52145), *cps* mutant (52145-Δ*wca*_K2_), or control strain (*K. aerogenes*) to resist predation by *D. discoideum* was tested on HL5-agar, pure or diluted. 1,000 amoebae were deposited on the bacterial lawns and plaques were recorded 5 days later. (C) The ability of *Dictyostelium* to grow on a bacterial lawn was assessed by depositing amoebae (from 10 to 10,000) on a lawn of bacteria grown on HL5–5% agar medium. A phagocytosis plaque was observed 5 days later when bacteria were permissive (*K. aerogenes* and 52145-Δ*wca*_K2_; *Δwca*_K2_).

We evaluated the resistance of the highly virulent clinical isolate *K. pneumoniae* strain 52145 (hereafter Kp52145) to predation by *D. discoideum* in comparison to the previously analyzed *K. aerogenes* susceptible strain, by using SM medium. Further confirming previous results [42], amoebae feed only upon *K. aerogenes*, creating phagocytic plaques (Figure 1A). Since CPS reduces phagocytosis, the lack of growth of *Dyctiostelium* on Kp52145 may simply reflect that these bacteria are not ingested by the amoebae. Unexpectedly, the isogenic *cps* mutant, strain 52145-Δ*wca*_K2_, was also not permissive for *Dictyostelium* growth (Figure 1A). This might be due to the fact that the CPS of this *Klebsiella* strain, of the K2 serotype, is not required for phagocytosis resistance. However, there are studies showing that indeed CPSs of the K2 serotype mediate resistance to phagocytosis [18], [48], [49]. Another possibility could be that the assay conditions were too favourable for Kp52145. In fact, a similar scenario was reported when the virulence of *Aeromonas spp* was analyzed using *D. discoideum* model [50], i.e. *Dictyostelium* was incapable of growing on wild-type bacteria or on any of the nonvirulent mutants of *Aeromonas* tested when the assays were performed on SM medium. Since the richness of the growth medium affects the threshold at which a bacteria is permissive for *Dictyostelium* [42], we tested a range of dilutions of HL5 to determine the medium where only Kp52145 remains nonpermissive (Figure 1B). We observed that 52145-Δ*wca*_K2_, but not Kp52145, was susceptible to predation even by 10 amoebae at 5% HL5 (Figure 1C). Kp52145 and 52145-Δ*wca*_K2_ exhibited similar growth rates in 5% HL5 (data not shown). Control experiments indicated that Kp52145 was not cytotoxic for *Dictyostelium* because the number of amoebae after incubation with Kp52145 was similar to that after incubation with *K. aerogenes* (1.6±0.5×10^5^, versus 1.7±0.6×10^5^; respectively, *P*>0.05).

**Figure 2:**
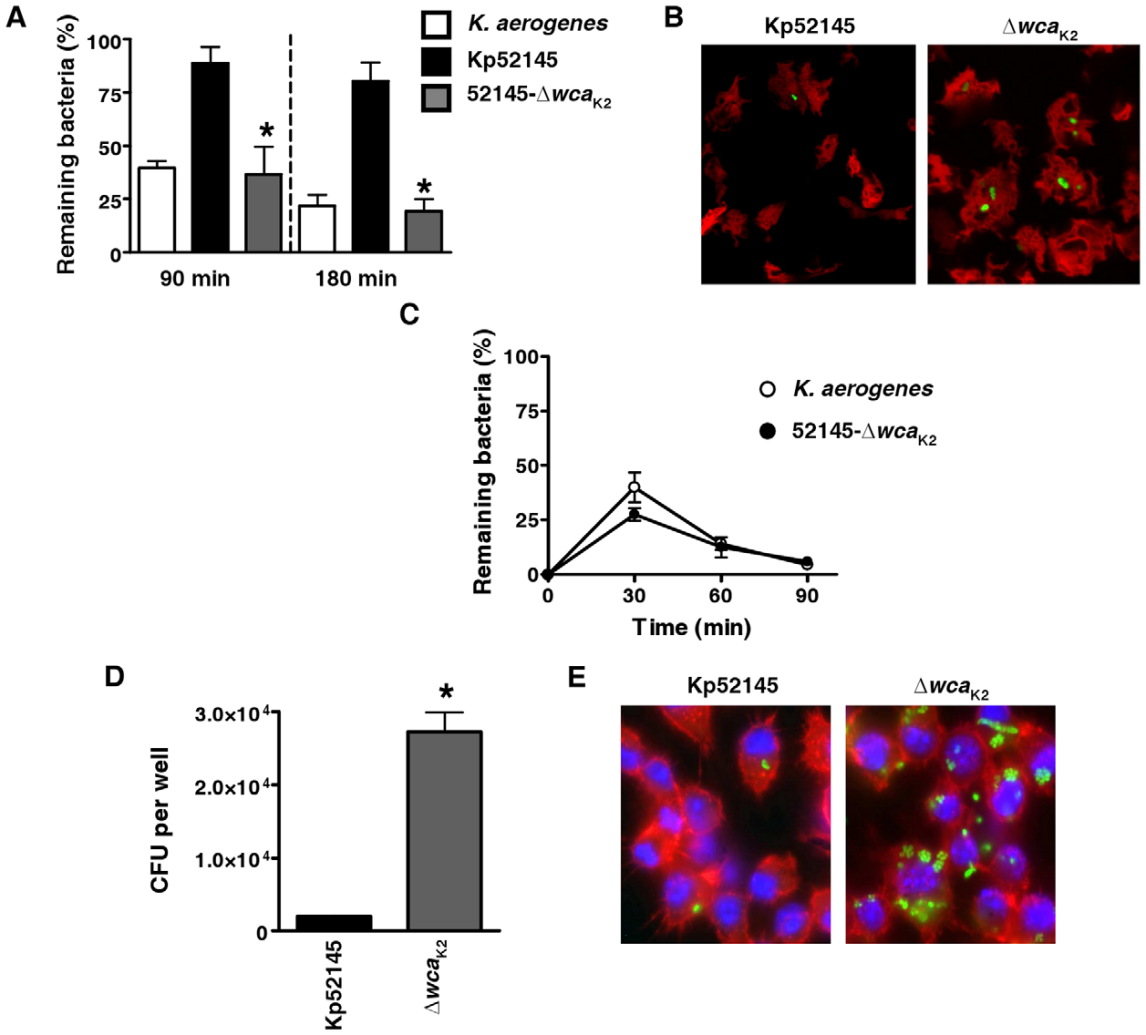
Role of *K. pneumoniae* CPS on phagocytosis resistance. (A) *Dictyostelium* cells were incubated with *Klebsiella* and the number of surviving bacteria (total or cell-associated) was determined at different times by killing the *Dictyostelium* and plating the bacteria on LB plates. Bars represent mean ± s.e.m (n = 4). *; *P*<0.05 (results are significantly different from the results for the wild-type strain [Kp52145]; two tailed *t* test). (B) *Dictyostelium* AX2/RFP cells were incubated in the presence of *Klebsiella* strains, *K. pneumoniae* (Kp52145), *cps* mutant (52145-Δ*wca*_K2_; Δ*wca*_K2_), containing plasmid pFPV25.1Cm for 30 min, and then fixed. Images are representative of three independent experiments. (C) *Dictyostelium* cells were incubated with *Klebsiella* and the number of intracellular bacteria was determined at different times by killing the *Dictyostelium* and plating the bacteria on LB plates. Each point represents the mean and standard deviation of twelve samples from four independent experiments. (D) *Klebsiella* phagocytois by MH-S mouse alveolar macrophages. Intracellular bacteria were determined by the gentamicin protection assay. Bars represent mean ± s.e.m (n = 4). *; *P*<0.05 (results are significantly different from the results for the wild-type strain [Kp52145]; two tailed *t* test). (E) Immunofluorescence confocal microscopy of MH-S mouse alveolar macrophages infected with *Klebsiella* strains Kp52145 or 52145-Δ*wca*_K2_ (Δ*wca*_K2_), containing plasmid pFPV25.1Cm. Actin cytoskeleton was stained with Phalloidin-RRX (red) and host cell nuclei were stained with Hoechst (blue). Images are representative of five independent experiments.

Survival experiments were carried out to determine the total number of remaining bacteria, as well as the number of live cell-associated bacteria. *Dictyostelium* ingested and killed 52145-Δ*wca*_K2_ as fast as the *K. aerogenes* control strain (Figure 2A). The number of intracellular 52145-Δ*wca*_K2_ was higher than that of intracellular Kp52145 after co-culture of bacteria and *Dictyostelium* with an average of 2 bacteria per infected amoeba (Figure 2B). The percentage of infected *Dyctiostelium* with Kp52145 was 7±3% whereas it reached 65±9% when the challenging strain was 52145-Δ*wca*_K2_. Altogether, these findings indicate that 52145-Δ*wca*_K2_ is more easily engulfed by *Dictyostelium* than Kp52145. Intracellular killing was so fast that viable intracellular bacteria were hardly detectable already at 60 min post infection (Figure 2C), suggesting that under these experimental conditions the limiting factor for killing was the rate of phagocytosis.

Gentamicin protection assays showed that 52145-Δ*wca*_K2_ was ingested by MH-S alveolar macrophages in higher numbers than the wild-type strain (Figure 2D). This correlated with the microscopic observation of higher numbers of intracellular 52145-Δ*wca*_K2_ than Kp52145 after co-culture with alveolar macrophages with an average of 4 bacteria per infected macrophage (Figure 2E). The percentage of macrophages infected with 52145-Δ*wca*_K2_ was significantly higher than that of macrophages infected with Kp52145 (43±9% and 9±3%, respectively; *P*<0.05).

Collectively, these results highlight the role of *K. pneumoniae* CPS of K2 serotype in resistance to phagocytosis by *D. discoideum* and alveolar macrophages.

### Role of *K. pneumoniae* LPS polysaccharide section on phagocytosis resistance

**Figure 3:**
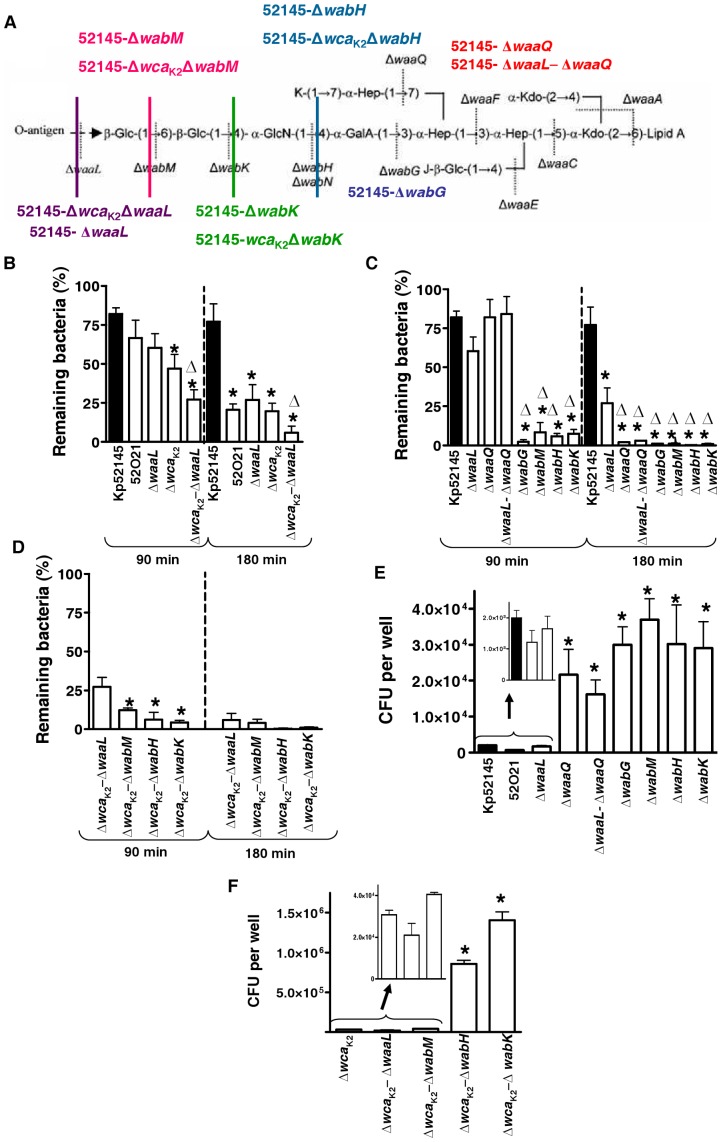
Role of *K. pneumoniae* LPS polysaccharide section on phagocytosis resistance. (A) *K. pneumoniae* 52145 (Kp52145) oligosaccharide structure based on a published study [35]. Lines denote the truncation level for the different core biosynthetic gene mutations. Residues J and K could be hydrogen (H) or GalA. (B) *Dictyostelium* cells were incubated with *Klebsiella* strains (wild type [Kp52145], LPS OPS mutants [52O21] and 52145-Δ*waaL* [Δ*waaL*], CPS mutant 52145-*Δwca*_K2_ [*Δwca*_K2_], or strain lacking the CPS and the OPS 52145-*Δwca*_K2_-*ΔwaaL* [*Δwca*_K2_-*ΔwaaL*]) and the number of surviving bacteria (total or cell-associated) was determined at different times by killing the *Dictyostelium* and plating the bacteria on LB plates. Bars represent mean ± s.e.m (n = 4). *; *P*<0.05 (results are significantly different from the results for the wild-type strain [Kp52145]; two tailed *t* test). ▵; *P*<0.05 (results are significantly different from the results for the *cps* mutant [52145-*Δwca*_K2_]; two tailed *t* test). (C) *Dictyostelium* cells were incubated with *Klebsiella* strains (wild type [Kp52145], LPS OPS mutant 52145-Δ*waaL* [Δ*waaL*], and LPS core mutants 52145-Δ*waaQ* [Δ*waaQ*], 52145-Δ*waaL-*Δ*waaQ* [Δ*waaL-*Δ*waaQ*], 52145-Δ*wabG* [Δ*wabG*] 52145-Δ*wabM* [Δ*wabM*], 52145-Δ*wabH* [Δ*wabH*], and 52145-Δ*wabK* [Δ*wabK*]) and the number of surviving bacteria was determined at different times by killing the *Dictyostelium* and plating the bacteria on LB plates. Bars represent mean ± s.e.m (n = 4). *; *P*<0.05 (results are significantly different from the results for the wild-type strain [Kp52145]; two tailed *t* test). ▵; *P*<0.05 (results are significantly different from the results for the *waaL* mutant [52145-Δ*waaL*]; two tailed *t* test). (D) *Dictyostelium* cells were incubated with *Klebsiella* mutant lacking the CPS and the OPS 52145-*Δwca*_K2_-Δ*waaL* (*Δwca*_K2_-Δ*waaL*), or the OPS and the first, second or third sugar of the core (strains 52145-Δ*wca*_K2_-Δ*wabM* [Δ*wca*_K2_-Δ*wabM*], 52145-*Δwca*_K2_-Δ*wabH* [Δ*wca*_K2_-Δ*wabH*], and 52145-Δ*wca*_K2_-Δ*wabK* [*Δwca*_K2_-Δ*wabK*] respectively). The number of surviving bacteria was determined at different times by killing the *Dictyostelium* and plating the bacteria on LB plates. Bars represent mean ± s.e.m (n = 4). *; *P*<0.05 (results are significantly different from the results for 52145-*Δwca*_K2_-Δ*waaL*; two tailed *t* test). (E) Phagocytosis of LPS polysaccharide mutants by MH-S mouse alveolar macrophages. Intracellular bacteria were determined by the gentamicin protection assay. Kp52145 (wild type); OPS mutants 52O21 and Δ*waaL* (52145-Δ*waaL*); LPS core mutants Δ*waaQ* (52145-Δ*waaQ*), Δ*waaL-*Δ*waaQ* (52145-Δ*waaL-*Δ*waaQ*), Δ*wabG* (52145-Δ*wabG*), Δ*wabM* (52145-Δ*wabM*), Δ*wabH* (52145-Δ*wabH*), and Δ*wabK* (52145-Δ*wabK*). Bars represent mean ± s.e.m (n = 4). *; *P*<0.05 (results are significantly different from the results for the wild-type strain [Kp52145]; two tailed *t* test). (F) MH-S cells engulfment of *Klebsiella cps* mutant, strain 52145-*Δwca*_K2_ (*Δwca*_K2_), or strains lacking the CPS and the OPS 52145-*Δwca*_K2_-Δ*waaL* (*Δwca*_K2_-Δ*waaL*), or the OPS and the first, second or third sugar of the core (strains 52145-*Δwca*_K2_-Δ*wabM* [*Δwca*_K2_-Δ*wabM*], 52145-*Δwca*_K2_-Δ*wabH* [*Δwca*_K2_-Δ*wabH*], and 52145-*Δwca*_K2_-Δ*wabK* [-*Δwca*_K2_-Δ*wabK*] respectively). *; *P*<0.05 (results are significantly different from the results for the *cps* mutant [52145-*Δwca*_K2_]; two tailed *t* test).

We sought to determine whether the LPS OPS and core sections contribute to phagocytosis resistance by *D. discoideum* and alveolar macrophages. Strains 52O21 and 52145-Δ*waaL* are mutants in the OPS transporter and the OPS ligase that attaches the OPS to the LPS core, respectively [13], [34]. Both mutants do not express the OPS and they express similar levels of CPS than the wild type [13], [34]. After 90 min of co-culture with amoebae, no significant differences in survival were found between the OPS mutants, strains 52O21 and 52145-Δ*waaL*, and the wild type (Figure 3B). However, a 50% decreased in survival of the two mutants was observed after 180 min of incubation (Figure 3B). We investigated whether the absence of the LPS OPS also increases the phagocytosis of 52145-Δ*wca*_K2_ by *D. discoideium*. Indeed, this was the case (Figure 3B). The survival of the double mutant lacking *cps* and OPS, strain 52145-Δ*wca*_K2_-Δ*waaL*, was significantly lower than that of the *cps* mutant, 52145-Δ*wca*_K2_ already after 90 min of co-culture with the amoebae (Figure 3B).

To delineate the possible contribution of the LPS core to phagocytosis resistance, we first analyzed strains 52145-Δ*waaQ*, lacking one heptose and its attached variable residue, and 52145-Δ*wabG*, lacking the first four sugars of the LPS core (Figure 3A). Whereas 52145-Δ*waaQ* expresses OPS and similar levels of CPS than the wild-type strain [51], [52], 52145-Δ*wabG* is devoid of cell-surface attached CPS and OPS [51]. Results shown in Figure 3C revealed that 52145-Δ*wabG* was more susceptible to predation by *D. discoideum* than 52145-Δ*waaQ*, which, in turn, was as susceptible as the OPS mutants. We did not observe significant differences between 52145-Δ*waaQ* and 52145-Δ*waaL*-Δ*waaQ* (Figure 3C). Since 52145-Δ*wabG* lacks the cell-surface attached CPS, the OPS and the sugars of the outer core region we analyzed other core mutants expressing CPS and a less truncated core. 52145-Δ*wabM*, 52145-Δ*wabK* and 52145-Δ*wabH*, lack, in addition to the OPS, the first, second and third sugar of the LPS core, respectively (Figure 3A), but expressed similar levels of CPS than the wild-type strain [35]. Results shown in Figure 3C revealed that 52145-Δ*wabM*; 52145-Δ*wabK* and 52145-Δ*wabH* were susceptible to predation by *D. discoideum* already after 90 min of co-culture with amoebae (Figure 3C). No significant differences were observed between these mutants and 52145-Δ*wabG* (Figure 3C). In the genetic background of the *cps* mutant, the three core mutants were more susceptible to predation by the amoebae than the LPS OPS mutant but only after 90 min of co-culture (Figure 3D).

Next, we analyzed the susceptibility of this set of mutant strains to phagocytosis by alveolar macrophages. No differences were found between the phagocytosis of the LPS OPS mutants, 52O21 and 52145-Δ*waaL*, and that of the wild-type strain (Figure 3E). In contrast, all the LPS core mutants tested were ingested in higher numbers than the LPS OPS mutants and the wild-type strain (Figure 3E). In the genetic background of the *cps* mutant, only *wabK* and *wabH* mutants were internalized in higher numbers than the LPS OPS mutant by the alveolar macrophages (Figure 3F).

In summary, our findings indicate that, in addition to the CPS, *K. pneumoniae* LPS OPS and the core sugars participate in the resistance to predation by *D. discoideum*. In the case of alveolar macrophages, the LPS core plays a more prominent role than the LPS OPS in *K. pneumoniae* avoidance of phagocytosis.

### Role of *K. pneumoniae* LPS lipid A decorations on phagocytosis resistance

52145-Δ*pmrF*, 52145-Δ*pagP*GB and 52145-Δ*pmrF*-Δ*pagP*GB are mutant strains lacking lipid A species containing aminoarabinose, palmitate or both [26]. These mutants express the same levels of CPS than the wild type [26]. LPS analysis showed that these mutants expressed OPS (Figure S2).

**Figure 4:**
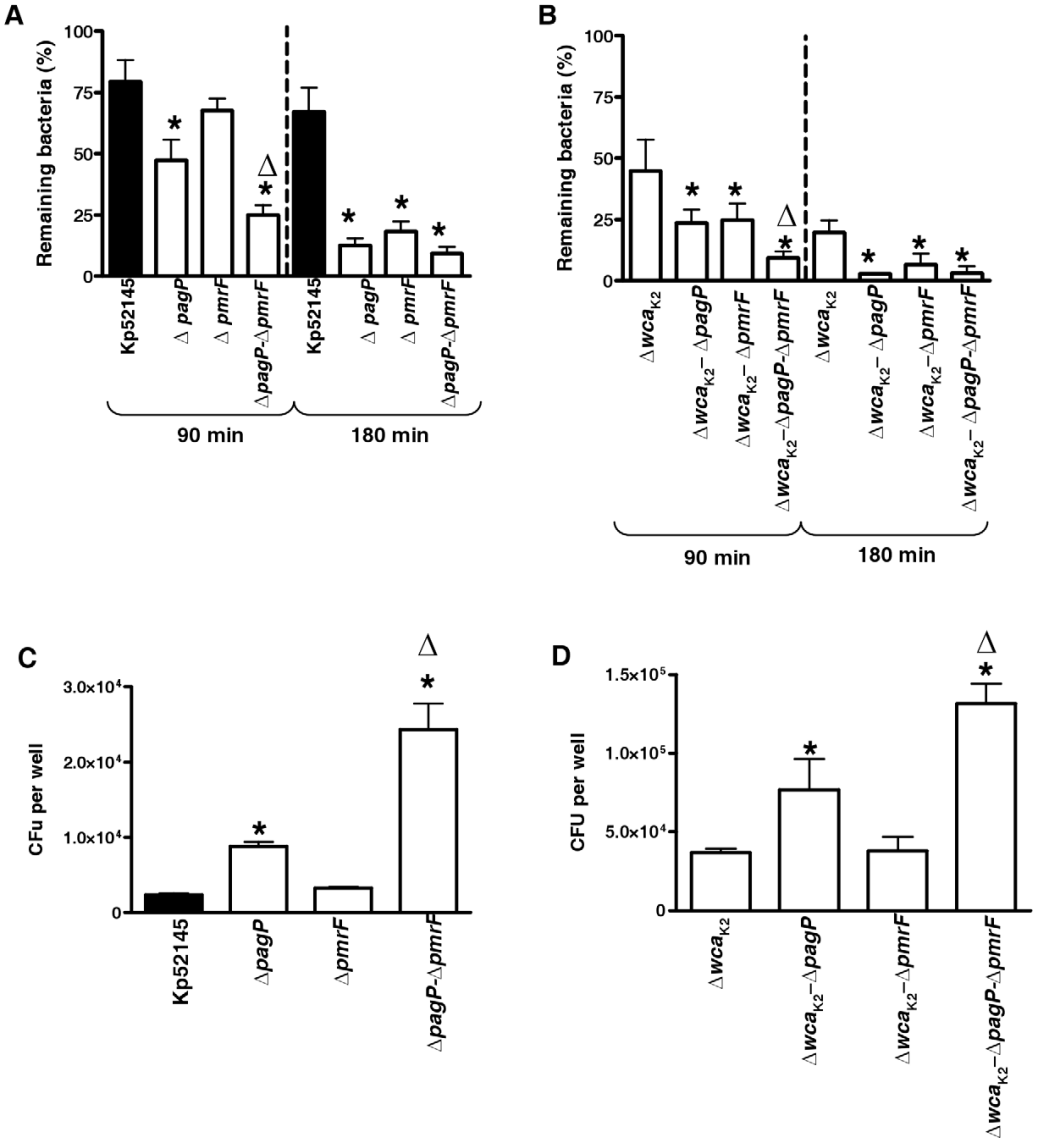
Role of *K. pneumoniae* lipid A decorations on phagocytosis resistance. (A) *Dictyostelium* cells were incubated with *Klebsiella* strains (wild type [Kp52145], *pagP* mutant (Δ*pagP*, 52145-Δ*pagP*GB), *pmrF* mutant (Δ*pmrF*, 52145-Δ*pmrF*), or *pagP-pmrF* double mutant (Δ*pagP*-Δ*pmrF*, 52145-Δ*pagP*GB-Δ*pmrF*). The number of surviving bacteria (total or cell-associated) was determined at different times by killing the *Dictyostelium* and plating the bacteria on LB plates. Bars represent mean ± s.e.m (n = 4). *; *P*<0.05 (results are significantly different from the results for the wild-type strain [Kp52145]; two tailed *t* test). ▵; *P*<0.05 (results are significantly different from the results for 52145-*ΔpagP*GB; two tailed *t* test). (B) *Dictyostelium* cells were incubated with *Klebsiella* lipid A mutants constructed in the background of the *cps* mutant (52145-*Δwca*_K2_ [*Δwca*_K2_]). The number of surviving bacteria was determined at different times by killing the *Dictyostelium* and plating the bacteria on LB plates. Bars represent mean ± s.e.m (n = 4). *; *P*<0.05 (results are significantly different from the results for the *cps* mutant [52145-*Δwca*_K2_]; two tailed *t* test). ▵; *P*<0.05 (results are significantly different from the results for 52145-Δ*wca*_K2_-*ΔpagP*GB; two tailed *t* test). (C) Phagocytosis of lipid A mutants by MH-S cells. Wild type [Kp52145], *pagP* mutant (Δ*pagP*, 52145-Δ*pagP*GB), *pmrF* mutant (Δ*pmrF*, 52145-Δ*pmrF*), or double *pagP-pmrF* mutant (Δ*pagP*-Δ*pmrF*, 52145-Δ*pagP*GB-Δ*pmrF*). Bars represent mean ± s.e.m (n = 4) *; *P*<0.05 (results are significantly different from the results for the wild-type strain [Kp52145]; two tailed *t* test). ▵; *P*<0.05 (results are significantly different from the results for 52145-*ΔpagP*GB; two tailed *t* test). (D) Phagocytosis of lipid A mutants constructed in the background of the *cps* mutant (52145-*Δwca*_K2_ [*Δwca*_K2_]) by MH-S cells. Bars represent mean ± s.e.m (n = 4). *; *P*<0.05 (results are significantly different from the results for the *cps* mutant [52145-*Δwca*_K2_]; two tailed *t* test). ▵; *P*<0.05 (results are significantly different from the results for 52145-*Δwca*_K2_-Δ*pagP*GB; two tailed *t* test).

We asked whether these modifications contribute to phagocytosis resistance by *D. discoideum*. After 90 min of co-culture, *pagP* mutant was more susceptible to predation by the amoebae than the wild type and the *pmrF* mutant whereas the *pagP-pmrF* double mutant was the most susceptible strain (Figure 4A). After 180 min, the three lipid A mutants were more susceptible to predation than the wild type and no significant differences between the mutant strains were observed (Figure 4A). In the background of the *cps* mutant, after 90 min of co-culture, the *pagP* and *pmrF* single mutants were more susceptible to predation than 52145-Δ*wca*_K2_ whereas 52145-Δ*wca*_K2_-Δ*pmrF*-Δ*pagP*GB triple mutant was the most susceptible strain (Figure 4B). After 180 min, the three lipid mutants were more susceptible than 52145-Δ*wca*_K2_ (Figure 4B).

Next, we assessed the contribution of lipid A decorations to phagocytosis resistance by alveolar macrophages. The *pagP* mutants, strains 52145-Δ*pagP*GB, 52145-Δ*pmrF-*Δ*pagP*GB, 52145-Δ*wca*_K2_-Δ*pagP*GB, and 52145-Δ*wca*_K2_-Δ*pmrF*-Δ*pagP*GB, were internalized in higher numbers by alveolar macrophages than Kp52145 and 52145-Δ*wca*_K2,_ being the numbers of 52145-Δ*wca*_K2_-Δ*pmrF*-Δ*pagP*GB the highest (Figure 4C-D).

Altogether, these data support the notion that the PagP-dependent lipid A modification with palmitate plays a more important role to reduce *K. pneumoniae* phagocytosis by *D. discoideum* and alveolar macrophages than the lipid A modification with aminoarabinose. The impact of the latter is more evident in the *pagP* mutant background.

### Role of *K. pneumoniae* OMPs on phagocytosis resistance

Previously, we have shown that *ompA* and *ompK36* mutants express similar levels of CPS than Kp52145 [30], [31]. We aimed to establish whether OmpA and OmpK36 are involved in the resistance to phagocytosis by *D. discoideum* and alveolar macrophages.

**Figure 5:**
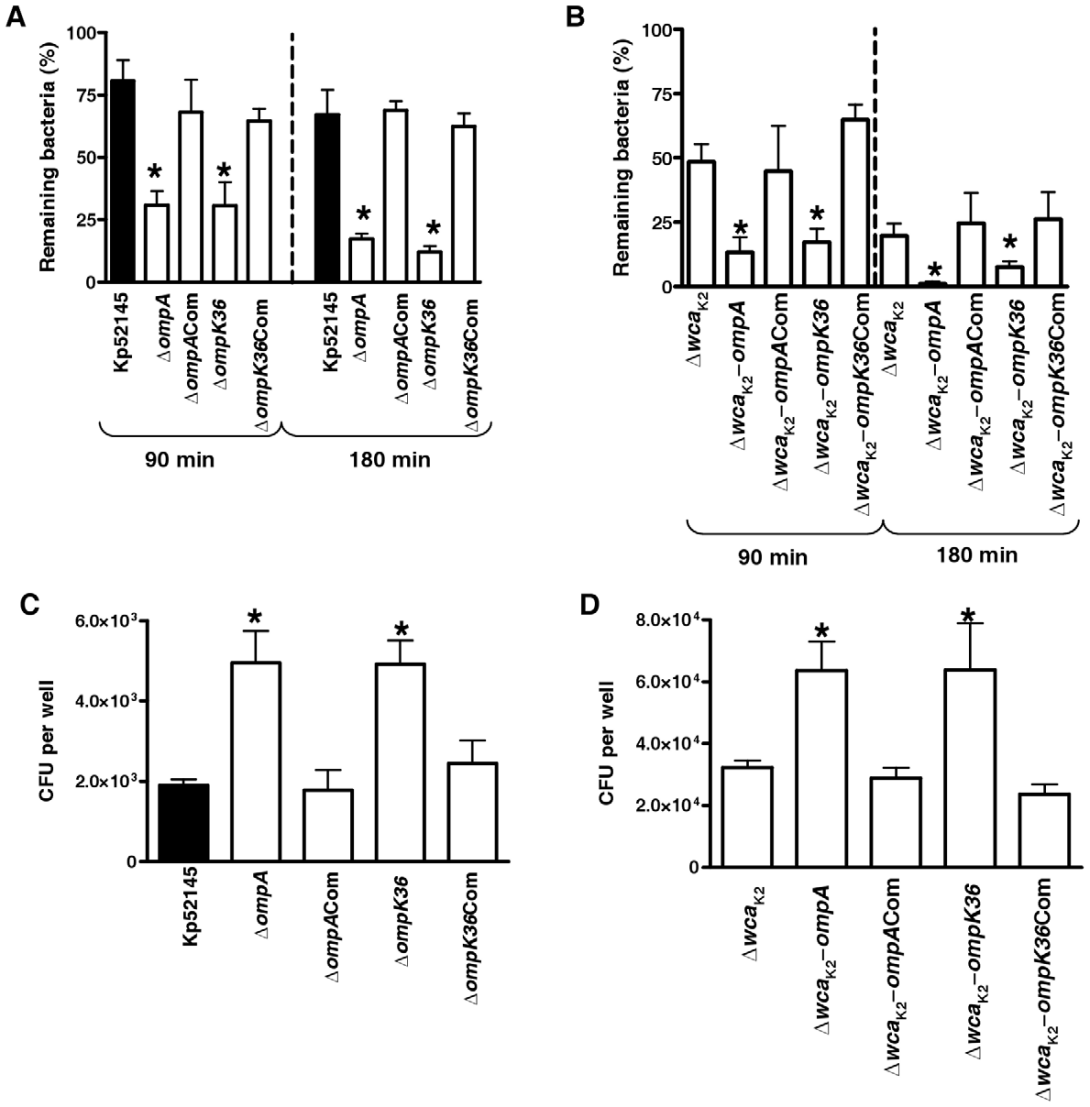
Role of *K. pneumoniae* OMPs on phagocytosis resistance. (A) *Dictyostelium* cells were incubated with *Klebsiella* strains (wild type [Kp52145], *ompA* mutant (Δ*ompA*, 52OmpA2), *ompK36* mutant (Δ*ompK36*, 52OmpK36), and the corresponding complemented strains. The number of surviving bacteria (total or cell-associated) was determined at different times by killing the *Dictyostelium* and plating the bacteria on LB plates. Bars represent mean ± s.e.m (n = 4). *; *P*<0.05 (results are significantly different from the results for the wild-type strain [Kp52145]; two tailed *t* test). (B) *Dictyostelium* cells were incubated with *Klebsiella* OMPs mutants constructed in the background of the *cps* mutant (52145-Δ*wca*_K2_, [Δ*wca*_K2_]). The number of surviving bacteria (total or cell-associated) was determined at different times by killing the *Dictyostelium* and plating the bacteria on LB plates. Bars represent mean ± s.e.m (n = 4). *; *P*<0.05 (results are significantly different from the results for the cps mutant [52145-Δ*wca*_K2_]; two tailed *t* test). (C) Phagocytosis of OMPs mutants by MH-S cells. Wild type [Kp52145], *ompA* mutant (Δ*ompA*, 52OmpA2), *ompK36* mutant (Δ*ompK36*, 52OmpK36), and the corresponding complemented strains. Bars represent mean ± s.e.m (n = 4) *; *P*<0.05 (results are significantly different from the results for the wild-type strain [Kp52145]; two tailed *t* test). (D) Phagocytosis of OMPs mutants constructed in the background of the *cps* mutant (52145-Δ*wca*_K2_) by MH-S cells. Bars represent mean ± s.e.m (n = 4). *; *P*<0.05 (results are significantly different from the results for the cps mutant [52145-Δ*wca*_K2_]; two tailed *t* test).

Results displayed in Figure 5A indicate that *ompA* and *ompK36* mutants were susceptible to predation by amoebae. The contribution of both OMPs to resist predation by *D. discoideum* was also observed in the *cps* mutant background (Figure 5B). Likewise, OMPs mutants were phagocytosed by alveolar macrophages in higher numbers than Kp52145 and 52145-Δ*wca*_K2_ (Figure 5C–D). OMPs mutants could be complemented (Figure 5).

### Virulence of *K. pneumoniae ompK*36 mutant

**Figure 6:**
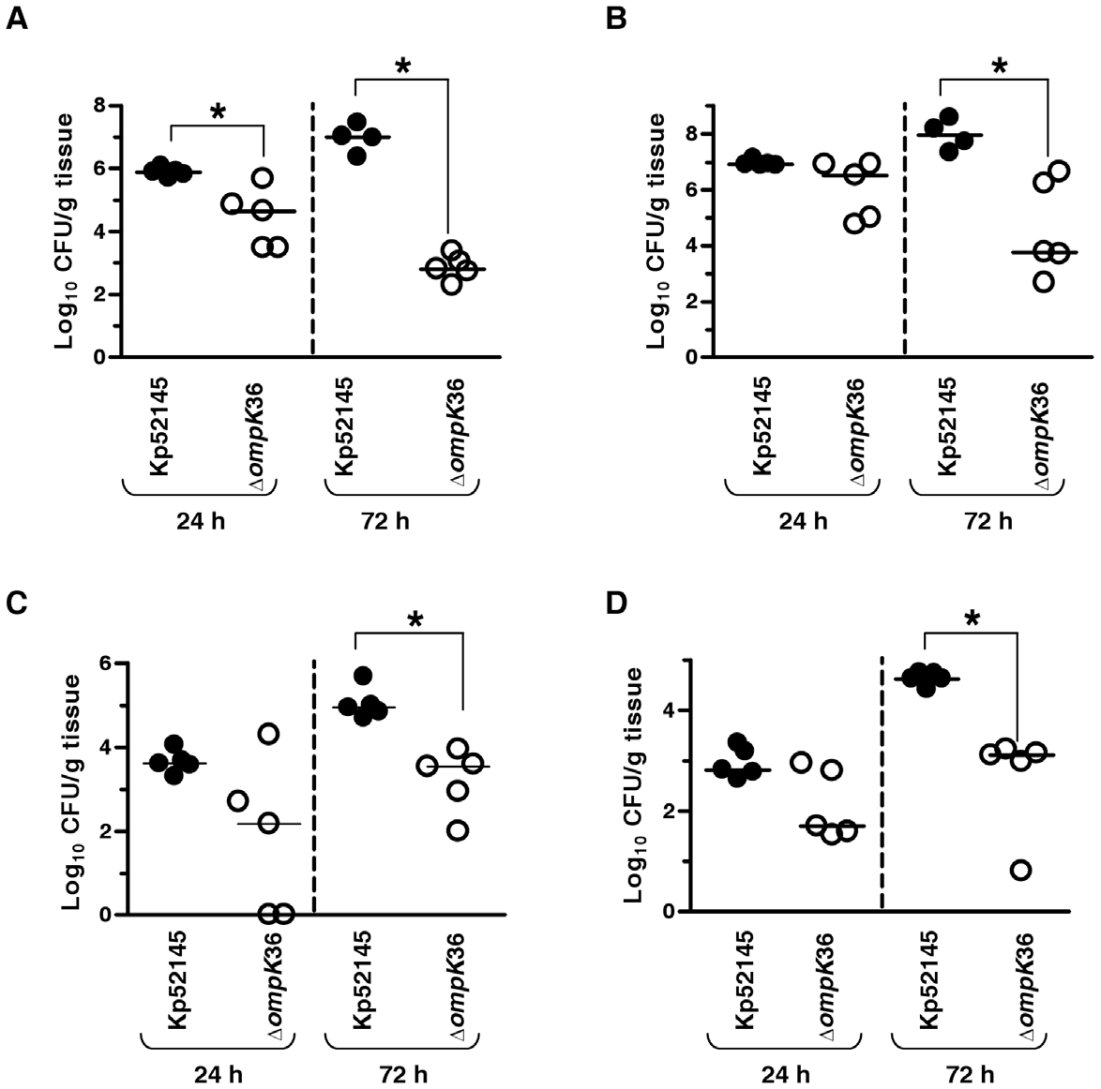
Virulence of *K. pneumoniae ompK36* mutant. Bacterial counts in mouse organs at 24 h post infection or 72 h post infection. Mice were infected intranasally with a bacterial mixture containing 5×10^4^ bacteria of wild type (Kp52145, •) or *ompK36* mutant (Δ*ompK36*, ○) Results were reported as log CFU per gram of tissue (Log CFU/g). *, results are significantly different (*P*<0.05; two-tailed *t* test) from the results for Kp52145. (A) Trachea; (B) Lung; (C) Spleen, (D) Liver.

We and others have assessed the contribution of CPS, LPS polysaccharides, lipid A decorations and *ompA* to *K. pneumoniae* virulence [13], [18], [26], [31], [34], [35], [53]. Notably, there is a strong correlation between resistance to phagocytosis (this work) and attenuation *in vivo*. Therefore, the fact that OmpK36 contributed to phagocytosis resistance prompted us to determine the ability of the *ompK*36 mutant to cause pneumonia. C57BL/6JOlaHsd mice were infected intranasally and bacterial loads in trachea and lung homogenates were determined at 24 and 72 h post-infection (Figure 6). Kp52145 and *ompK36* mutant colonized trachea although bacterial loads of the mutant were lower than those of the wild type at 24 and 72 h post-infection (Figure 6A). *ompK36* mutant also colonized the lungs and at 72 h post infection bacterial loads of the mutant were lower than those of the wild type (Figure 6B).

To evaluate the ability of the mutant to disseminate to other organs, bacterial loads in spleen and liver were determined. *ompK36* mutant reached both organs (Figure 6C–D) and at 72 h post-infection the bacterial loads in spleen and liver were significantly lower than those of the wild type (Figure 6C–D).

## Discussion

Phagocytosis is one of the key processes of the immune system. Although most bacteria are successfully internalized and eliminated by phagocytes, several pathogens have developed survival strategies that interfere with the internalization and/or maturation processes. *K. pneumoniae* is a well known example of one pathogen displaying resistance to phagocytosis. Prevention and management of the infections caused by such pathogens would obviously benefit from understanding the manner in which they circumvent and often co-opt the immune response. The fact that *K. pneumoniae* is ubiquitous in nature and, therefore, should avoid predation by protozoa, including amoebae, poses the question whether *K. pneumoniae* employs similar means to counteract predation by amoebae and engulfment by mammalian phagocytes. In this study, we provide evidence for this notion. Furthermore, our data reveal novel information about the implication of *K. pneumoniae* LPS polysaccharide and lipid A sections, and of the OMPs OmpA and OmpK36 to *K. pneumoniae* avoidance of phagocytosis.

In this study, we found a correlation between the findings obtained testing alveolar macrophages, key cells responsible for lung defence against infections, and those found challenging *D. discoideum*. Therefore, our results add further evidence to the notion that *D. discoideum* model is useful for investigating phagocytosis. Our data further support the idea that the limiting factor for killing *Klebsiella* by the amoebae is the rate of phagocytosis [17], [44] since bacterial survival was not affected in those strains not engulfed by *D. discoideum*. Nevertheless, our results also highlight the importance of adjusting the assay conditions in order to set the threshold of the assay for supposedly permissive bacteria, in our case the *cps* mutant [17]–[19]. This was so even for a bacterial species previously tested, *K. pneumoniae*, thereby suggesting that the interplay between *D. discoideum* and bacterial pathogens is strain specific. Likewise, not all strains of *Vibrio cholerae* are able to avoid predation by *D. discoideum* [54]. The assay established and used in this study allowed a comprehensive analysis of *Klebsiella* surface determinants mediating resistance to phagocytosis with a high degree of reproducibility.

While this work was in progress, Pan and co-workers [19] reported the results of a screening to identify *K. pneumonia* NHTU-K2044 determinants preventing predation by *D. discoideum*. Seventy two of the mutants permissive for *D. discoideum* growth had transposon insertions in the *cps* operon [19], which is in good agreement with our findings showing the importance of CPS to resist phagocytosis. Of note, Kp52145 and NHTU-K2044 express CPS of different K serotypes, K2 and K1 respectively, thereby suggesting that the CPS serotype may not be the determinant factor mediating CPS-dependent reduction of phagocytosis. In fact, evidence points out that the critical factor is the amount of CPS expressed [19], [55]. Like most clinical isolates associated to severe infections, Kp52145 and NHTU-K2044 are heavily capsulated strains.

Twenty one of the NHTU-K2044 mutants supporting the growth of the amoebae had transposon insertions affecting the biosynthesis of the LPS OPS [19]. However, those mutants were less capsulated than the wild type [19] making difficult to delineate the relative contribution of the OPS and CPS to resist predation by *D. discoideum*. The OPS mutants tested in this work express wild-type levels of CPS hence allowing us to study the role of OPS on resistance to phagocytosis by *D. discoideum*. Indeed, our data highlight that the OPS limits predation by *D. discoideum*. Unexpectedly, the OPS mutants were phagocytosized by alveolar macrophages in similar numbers than the wild-type strain. Likewise, it has been reported no role for *Klebsiella* OPS on the resistance to phagocytosis by human dendritic cells [56]. In contrast, it has been shown that the OPS does play a role in the interaction with mouse and human neutrophils [19], [57]. Therefore, it is tempting to postulate that the contribution of *Klebsiella* OPS to prevent phagocytosis is not uniform to all professional phagocytes.

The contribution of LPS core to virulence is poorly characterized in most Gram negative pathogens and it has been only conclusively established for *Yersinia enterocolitica* and Kp52145 [35], [58]. To the best of our knowledge, our study is the first one highlighting the contribution of LPS core residues to phagocytosis resistance. Our findings showed that the heptose branch linked to the core by WaaQ is implicated in resistance to phagocytosis by *D. discoideum* and alveolar macrophages even in the presence of OPS. To investigate the contribution of other core residues in an OPS-bearing strain, we have used defined mutants that lack the OPS in addition to core residues which, in turn, suggest that the core residues are never exposed in a wild-type strain. However, it should be noted that epidemiological data indicate that nearly 10% of *Klebsiella* clinical isolates do not express the LPS OPS [59] and, therefore, core residues will not be masked by the OPS. Our results revealed that the first glucose residue of the LPS core is necessary to avoid engulfment by *D. discoideum* and alveolar macrophages since the relative survival of *wabM* mutant, lacking also OPS, was lower than those of the OPS mutants. Elimination of additional core residues did not further decrease the observed phagocytosis resistance.

Perusal of the literature clearly shows the importance of lipid A decorations with aminoarabinose and palmitate to counteract the microbial action of antimicrobial peptides. However, the role of these lipid A decorations, if any, to resist phagocytosis was unknown. Our data indicated that PagP-dependent lipid A palmitoylation plays an important role to reduce *Klebsiella* engulfment by *D. discoideum* and alveolar macrophages. Intriguing, a *pagP*-like gene also confers *Legionella pneumophila* resistance to antimicrobial peptides and contributes to the intracellular life of the pathogen in *Hartmannella vermiformis* amoebae and human macrophages [60]. It is tempting to formulate that PagP-dependent lipid A modification is a major bacterial determinant against the soluble and the cellular arms of the innate immune system. As a consequence, *pagP* mutants should be attenuated as indeed it has been indeed shown for *K. pneumoniae* and *Legionella* [26], [60]. Studies in other bacterial models are required to further validate our hypothesis.

Finally, we showed that *Klebsiella* OMPs also contribute to phagocytosis resistance in *K. pneumoniae*. Mounting evidence indicates that an essential attribute of *K. pneumoniae* OmpA is to thwart the innate immune system [30], [31]. The findings reported in this work further corroborate this notion and add new features to the previously described panoply of OmpA-dependent anti-immune strategies. In turn, the possible role of OmpK36 on *K. pneumoniae* evasion of innate immunity is poorly characterized. Our results revealed that OmpK36 also contributes to phagocytosis resistance by *Klebsiella*. However, and in contrast to OmpA, OmpK36 does not play any role in the resistance to antimicrobial peptides [30] and therefore it seems that both OMPs are not functionally redundant in terms of immune evasion. We are currently assessing whether OmpK36 modulates the cellular responses upon *Klebsiella* infection.

Previous reports suggested that alveolar macrophages play a major role in host defence against *K. pneumoniae* since the depletion of these cells results in reduced killing of the pathogen *in vivo* [10], [11]. Conversely, this suggests that *Klebsiella* countermeasures against phagocytosis could be important virulence factors. Data reported in this work give experimental support to this hypothesis since we have found a nearly perfect correlation between virulence using the pneumonia mouse model and resistance to phagocytosis by *D. discoideum* and alveolar macrophages. Thus, *cps*, *pagP*, LPS core, *ompA* and *ompK36* mutants are attenuated *in vivo* (this work and [13], [18], [26], [31], [34], [35], [53]) and in this study we have demonstrated that these loci mediate phagocytosis resistance. A tantalizing observation is that *Dictyostelium* amoebae might be useful as host model to measure *K. pneumoniae* virulence and not only phagocytosis. Given the relatively simplicity and low cost to create banks of *D. discoideum* mutants in comparison to mouse or human macrophages, it can be envisaged a more systematic analysis of the complex interactions between *Klebsiella* and the host, aiming to identify host resistance genes. First attempts challenging a non-saturating *D. discoideum* library of mutants with a laboratory adapted *K. pneumoniae* strain led to the identification of a type V P-ATPAase as an essential element for killing of *Klebsiella* [44]. Interestingly, this P-ATPAase was dispensable for the elimination of other bacteria [44] hence suggesting that *Klebsiella* may mobilize a specific set of host gene products only necessary for resistance to *K. pneumoniae* infection.

## Supporting Information

Figure S1 **Analysis of OMPs from** ***Klebsiella*** **strains.** SDS-PAGE (the acrylamide concentration was 4% in the stacking gel and 12% in the separation one) followed by Coomasie brilliant blue staining of OMPs from (A) Kp52145, 52OmpK36 and 52OmpK36Com; and (B) 52145-Δ*wca*_K2_, 52145-Δ*wca*_K2_-ompK36 and 52145-Δ*wca*_K2_-ompK36Com. MW, molecular weight marker.
(TIF)

Figure S2 **Analysis of LPSs from** ***Klebsiella*** **strains.** SDS-PAGE (the acrylamide concentration was 4% in the stacking gel and 12% in the separation one) followed by staining using Pro-Q Emerald 300 Lipopolysaccharide Gel Stain Kit (Invitrogen) of LPSs from Kp52145, 52145-Δ*pmrF* (Δ*pmrF*), 52145-Δ*pagP*GB (Δ*pagP*) and 52145-Δ*pagP*GB-Δ*pmrF* (Δ*pagP-*Δ*pmrF*).
(TIF)
