# Supplementary figures and images for: Role of Bacterial Surface Structures on the Interaction of Klebsiella pneumoniae with Phagocytes
Source: PLoS One. 2013 Feb 15;8(2):e56847. doi: 10.1371/journal.pone.0056847 (PMC3574025; doi:10.1371/journal.pone.0056847)

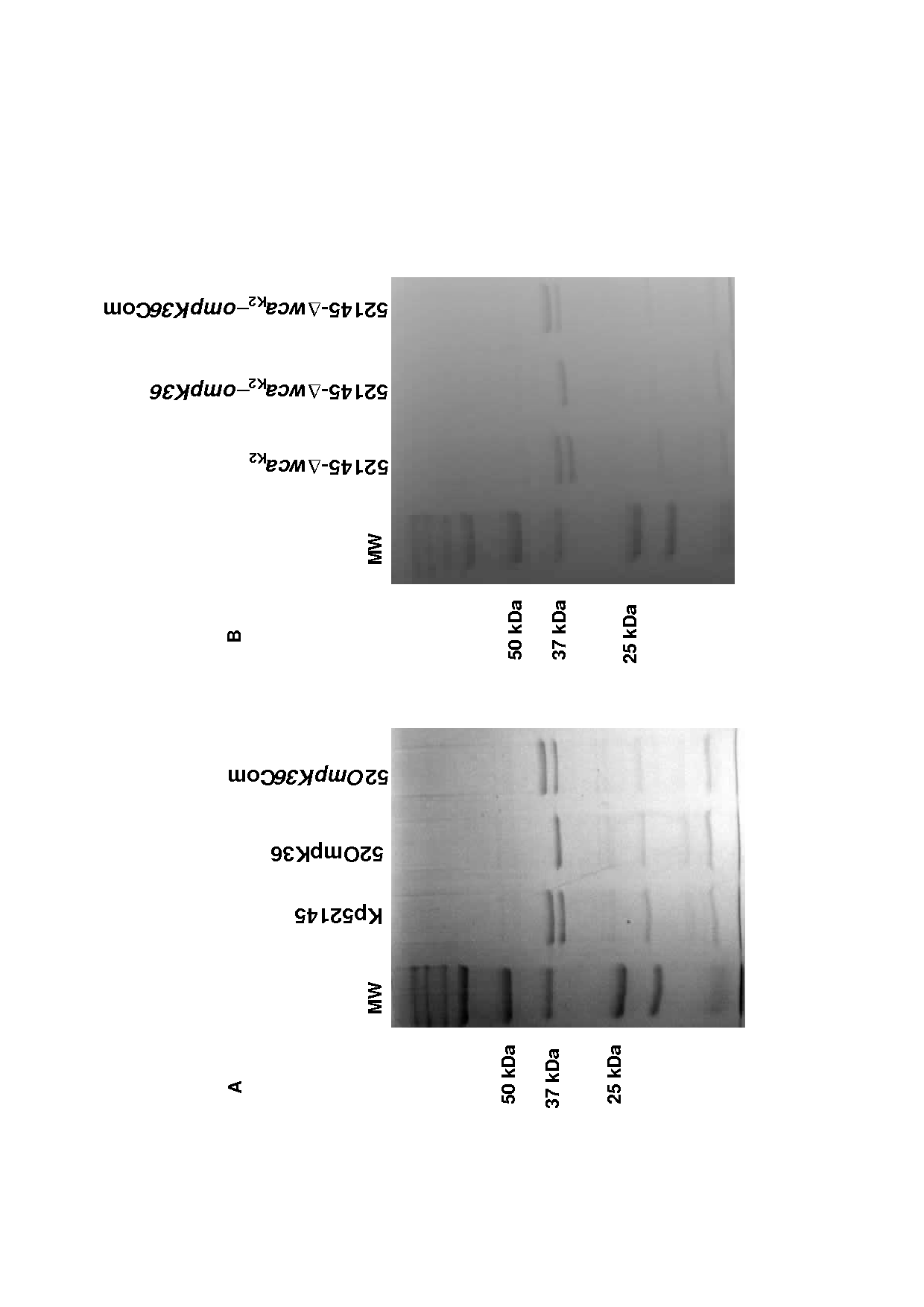

Supplement: Figure S1 — Analysis of OMPs from Klebsiella strains. SDS-PAGE (the acrylamide concentration was 4% in the stacking gel and 12% in the separation one) followed by Coomasie brilliant blue staining of OMPs from (A) Kp52145, 52OmpK36 and 52OmpK36Com; and (B) 52145-Δwca K2, 52145-Δwca K2-ompK36 and 52145-Δwca K2-ompK36Com. MW, molecular weight marker. (TIF) [file pone.0056847.s001.tif]

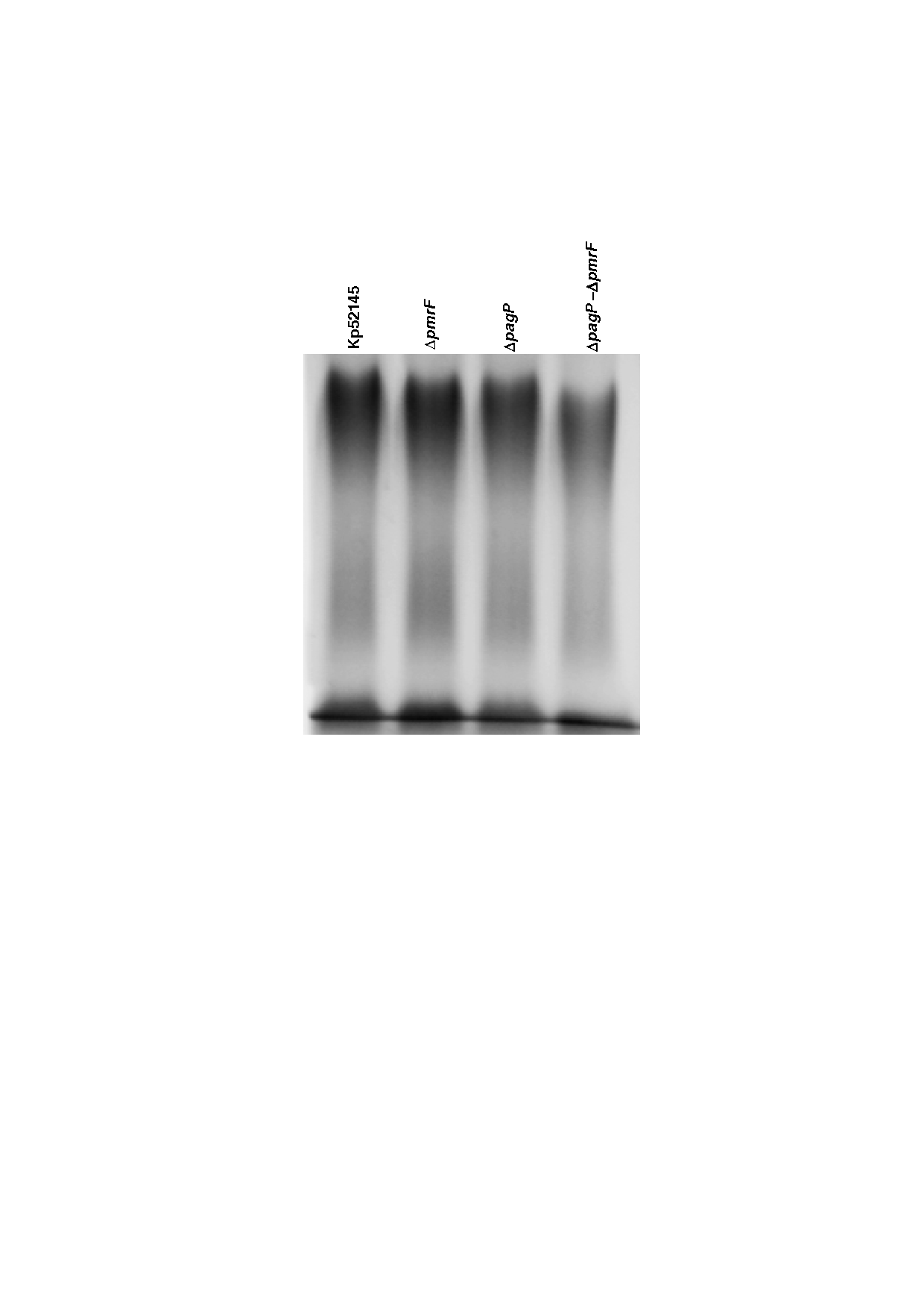

Supplement: Figure S2 — Analysis of LPSs from Klebsiella strains. SDS-PAGE (the acrylamide concentration was 4% in the stacking gel and 12% in the separation one) followed by staining using Pro-Q Emerald 300 Lipopolysaccharide Gel Stain Kit (Invitrogen) of LPSs from Kp52145, 52145-ΔpmrF (ΔpmrF), 52145-ΔpagPGB (ΔpagP) and 52145-ΔpagPGB-ΔpmrF (ΔpagP-ΔpmrF). (TIF) [file pone.0056847.s002.tif]
